# Diagnostic performance of automated, streamlined, daily updated exome analysis in patients with neurodevelopmental delay

**DOI:** 10.1186/s10020-022-00464-x

**Published:** 2022-03-26

**Authors:** Go Hun Seo, Hane Lee, Jungsul Lee, Heonjong Han, You Kyung Cho, Minji Kim, Yunha Choi, Jeongmin Choi, In Hee Choi, Seonkyeong Rhie, Kyu Young Chae, Yoo-Mi Kim, Chong Kun Cheon, Su Jin Kim, Jieun Lee, Eungu Kang, Jung Hye Byeon, Hee Joon Yu, Young-Lim Shin, Arum Oh, Woo Jin Kim, Mi-Sun Yum, Beom Hee Lee, Baik-Lin Eun

**Affiliations:** 1 3billion Inc., Seoul, South Korea; 2 Department of Pediatrics, Asan Medical Center Children’s Hospital, University of Ulsan College of Medicine, 88, Olympic-ro 43-Gil, Songpa-Gu, Seoul, 05505 South Korea; 3 Medical Genetics Center, Asan Medical Center, University of Ulsan College of Medicine, Seoul, South Korea; 4 Department of Pediatrics, CHA Bundang Medical Center, CHA University School of Medicine, Seongnam, South Korea; 5 Department of Pediatrics, Chungnam National University Sejong Hospital, Sejong, South Korea; 6 Department of Pediatrics, Pusan National University Children’s Hospital, Yangsan, Korea; 7 Department of Pediatrics, Inha University Hospital, Inha University College of Medicine, Incheon, South Korea; 8 Department of Pediatrics, Korea University Guro Hospital, 148 Gurodong-ro, Guro-gu, Seoul, 08308 South Korea; 9 Department of Pediatrics, Hallym University College of Medicine, Hwasung, South Korea; 10 Department of Pediatrics, Soonchunhyang University Bucheon Hospital, Soonchunhyang University School of Medicine, Bucheon, South Korea; 11 Department of Pediatrics, Chungbuk National University Hospital, Chungbuk National University College of Medicine, Cheongju, South Korea; 12 Department of Laboratory Medicine, EONE Laboratories, Seoul, South Korea

**Keywords:** Whole exome sequencing, Neurodevelopmental delay, Reanalysis

## Abstract

**Background:**

The diagnostic yield of whole-exome sequencing (WES) varies from 30%–50% among patients with mild to severe neurodevelopmental delay (NDD)/intellectual disability (ID). Routine retrospective reanalysis of undiagnosed patients has increased the total diagnostic yield by 10–15%. Here, we performed proband-only WES of 1065 patients with NDD/ID and applied a prospective, daily reanalysis automated pipeline to patients without clinically significant variants to facilitate diagnoses.

**Methods:**

The study included 1065 consecutive patients from 1056 nonconsanguineous unrelated families from 10 multimedical centers in South Korea between April 2018 and August 2021. WES data were analyzed daily using automatically updated databases with variant classification and symptom similarity scoring systems.

**Results:**

At the initial analysis, 402 patients from 1056 unrelated families (38.0%, 402/1,056 families) had a positive genetic diagnosis. Daily prospective, automated reanalysis resulted in the identification of 34 additional diagnostic variants in 31 patients (3%), which increased our molecular diagnostic yield to 41% (433/1056 families). Among these 31 patients, 26 were diagnosed with 23 different diseases that were newly discovered after 2019. The time interval between the first analysis and the molecular diagnosis by reanalysis was 1.2 ± 0.9 years, which was shorter in the patients enrolled during the latter part of the study period.

**Conclusion:**

Daily updated databases and reanalysis systems enhance the diagnostic performance in patients with NDD/ID, contributing to the rapid diagnosis of undiagnosed patients by applying the latest molecular genetic information.

**Supplementary Information:**

The online version contains supplementary material available at 10.1186/s10020-022-00464-x.

## Introduction

The prevalence of neurodevelopmental delay (NDD)/intellectual disability (ID) is estimated to be from 3% to 17% of children aged 3 to 17 years (Ropers 2010; Vasudevan and Suri 2017; Zablotsky et al. 2019). NDD/ID are phenotypically heterogeneous among patients in terms of the range and severity and can occur alone or in combination with multiple congenital anomalies, dysmorphic features, behavioral problems and additional neurological features such as epilepsy (Casanova et al. 2018; Lecoquierre et al. 2019). NDD/ID are also genetically heterogeneous, with more than 700 associated genes, making it difficult to pinpoint the causal gene; thus, many patients remain undiagnosed (Vissers et al. 2016; Study 2017).

Currently, chromosomal microarray (CMA) is recommended as the first-line diagnostic test for patients with NDD/ID to identify copy number variants such as deletions and duplications (Moeschler and Shevell 2014; Srour and Shevell 2014). However, the diagnostic yield of CMA is approximately 10–15%, and many NDD/ID patients remain undiagnosed (Clark et al. 2018). A significant reduction in the cost of next-generation sequencing (NGS) has led to widespread use of trio- or proband-based whole exome or genome sequencing for identifying causal variants among NDD/ID patients, and the diagnostic yield is higher compared to that of CMA (Vissers et al. 2016; Ligt et al. 2012; Rauch et al. 2012). The diagnostic yield of whole-exome sequencing (WES) varies from 30% to 50% among patients with mild to severe NDD/ID (Clark et al. 2018; Ligt et al. 2012), making WES an essential diagnostic tool for establishing a molecular diagnosis in children with NDD/ID (Clark et al. 2018; Srivastava et al. 2019). Routine reanalysis of data from undiagnosed patients often leads to a new diagnosis. The interval between the initial analysis and reanalysis typically ranges from 1 to 3 years, and among those undiagnosed patients for whom reanalysis was performed, the reported diagnostic rate ranges from 15–30%, increasing the total diagnostic yield by 10–15% (Liu et al. 2019; Costain et al. 2018). This increase in diagnostic rate can be attributed to identifying previously missed variants with advanced bioinformatics tools or collecting more informative phenotypic data from the patients; however, most of the diagnoses result from the discovery of new disease-causing genes (Liu et al. 2019; Fung et al. 2020).

In a previous single-center pilot study, we introduced a new automated, streamlined variant analysis software, referred to as EVIDENCE (Seo et al. 2020), which yielded a 42.7% diagnostic rate in patients with various clinical phenotypes. Here, we performed proband-only WES of 1065 consecutive patients with NDD/ID from 10 multimedical centers in South Korea. In this study, we implemented a daily reanalysis pipeline using EVIDENCE to enhance the diagnostic rate by quickly identifying a new gene-phenotype discovery or a change in variant pathogenicity.

## Methods

### Patients

The study enrolled an unselected series of 1065 affected individuals from 1056 nonconsanguineous families who were clinically suspected to have a genetic disorder. The patients were seen at one of the 10 clinics in South Korea from April 2018 to August 2021. Their detailed demographics, including age, sex, clinical diagnosis at the visit, family history, laboratory findings, radiologic findings, and genetic testing results, were reviewed.

Patients were included if they were strongly suspected by clinicians to have a genetic disease accompanied by neurodevelopmental delay, developmental regression, or intellectual disability. All patients were exome sequenced and analyzed as probands only after informed consent was obtained from the patients or their legal guardians after comprehensive genetic counseling. Patients and/or their guardians were counseled about the potential disclosure of medically actionable secondary findings according to the American College of Medical Genetics (ACMG) guidelines (v2.0) (Kalia et al. 2016), and they were given the option of receiving the information. The study was approved by the Institutional Review Board for Human Research of each medical center (IRB numbers: 2018‐0574, 2018‐0180, CHA-2018-06-008, CHH 2020-L06-01, 2020-05-040, 2020AS0186, 2020AN0332, 2020-03-031, 2020-08-003).

### Whole exome sequencing

Blood or buccal swab samples were collected from each patient, and genomic DNA was extracted from each sample. Most of the exonic regions of ~ 22,000 human genes were captured by one of the following 3 kits, depending on when the patient was enrolled: Agilent Sure Select kit (version C2, December 2018), Twist capture kit (Twist Bioscience HQ, San Francisco, CA, USA), or IDT xGen Exome Research Panel v2 (Integrated DNA Technologies, Coralville, Iowa, USA). Sequencing was performed using an Illumina NovaSeq6000 (San Diego, CA, USA) as 150 bp paired-end reads. The binary base call (BCL) sequence files generated by the NovaSeq6000 were converted and demultiplexed to FASTQ files. FASTQ files are aligned to the human reference genome (GRCh37/19 from NCBI, February 2009) to generate BAM files by BWA-MEM (v.0.7.17) (Li and Durbin 2009). Aligned BAM files were sorted and extracted using the statistical metric by samtools (v.1.9) (Li et al. 2009). Duplication was marked by Picard (v.2.20.8) (http://broadinstitute.github.io/picard/). Variant calling file were generated following the GATK best practices (GATK v.3.8) (McKenna et al. 2010). The mean depth of coverage was 125 X (> 20 X = 97%). Detail information about sequencing quality was noted in Additional file 1: Table S1.

### Variant analysis by EVIDENCE and daily reanalysis

Variants were annotated, filtered and prioritized using software developed in-house, EVIDENCE. EVIDENCE incorporates daily automatically updated databases, variant classification schema based on the ACMG guidelines, and a symptom similarity scoring system as previously described (Seo et al. 2020; Richards et al. 2015). Automatically updated databases include public databases, in-house variant databases and manually curated literature databases. The full list is shown in Additional file 2: Table S2. The variant classification schema incorporates the ACMG classification guidelines recommended by the ClinGen Sequence Variant Interpretation working group and assigns each variant a classification by weighing the strength of the evidence (Abou Tayoun et al. 2018; Harrison et al. 2019). The final assessment of the variant pathogenicity was determined manually by medical geneticists based on the clinical indications.

A daily reanalysis pipeline was implemented in April 2019 and patients without definite diagnosis were reanalyzed by October 2021. For patients with no clinically significant variants, only a uncertain significance of variant (VUS), potential compound heterozygous variants with one pathogenic (P)/likely pathogenic (LP) variant and one VUS, or multiple VUS reported, EVIDENCE was run on a daily basis with new annotations for the entire study period. If one or more variants were newly reclassified as P, LP or VUS with a symptom similarity score ≥ 5 that significantly increased the probability of being diagnosed compared to a score < 5, as described in our previous study (Seo et al. 2020), the suggested variant was rereviewed by medical geneticists and physicians. Since patient phenotypes can change over time, the pipeline updated the symptom similarity score when physicians submitted additional phenotype data after the initial analysis. Those variants excluded through the rereview process were not relisted until a new change was detected.

All identified variants in this study were confirmed by Sanger sequencing. A subset of the reported variants was analyzed in available family members by Sanger sequencing for segregation analysis.

### Reporting

A positive report consisted of one or more variants that were estimated to be the disease-causing variants as follows: for an autosomal dominant disease or an X-linked disease, one heterozygous or hemizygous P/LP variant in a known disease gene that would fit the phenotype and for an autosomal recessive disease, one homozygous P/LP variant, two P/LP compound heterozygous variants or potential compound heterozygous variants with one P/LP variant and a VUS with 2 moderate pathogenic criteria based on ACMG guidelines in a known disease gene that would fit the phenotype. An inconclusive report consisted of one heterozygous or hemizygous VUS in a known autosomal dominant or X-linked disease gene that would fit the phenotype well or potential compound heterozygous variants with one P/LP variant and one VUS with less than one moderate pathogenic criterion in a known autosomal recessive disease gene. If P/LP variants in a gene that had not been previously associated with disease defined in OMIM but reported in the literature, they were reported as inconclusive. In addition, if P/LP variants in known disease genes could fit the phenotype but needed additional phenotyping to confirm the phenotypic match, they were reported as inconclusive. Finally, if no clinically significant variant was found, a negative report was generated.

## Results

### Patient demographics

The demographic characteristics of the 1065 patients (585 males and 480 females) are shown in Table 1. The age at presentation ranged from less than age 1 month to 66 years. About eighty five percent of the patients were younger than 1 year at presentation, and 58.9% were younger than 4 months.

**Table 1:** Demographics of patients with neurodevelopmental delay/intellectual disability

Category	Number of patients (%)
Sex (male: female)	585 (54.9): 480 (45.1)
Onset at presentation	
Antenatal	10 (0.9%)
Neonatal (from birth to 4 months)	628 (58.9%)
Infant (from more than 4 months to1 year)	273 (25.6%)
Early childhood (from more than1 year to 5 years)	65 (6.1%)
Childhood (from more than 5 years to 12 years)	77 (7.2%)
Adolescent (from more than 12 years to 18 years)	10 (0.9%)
Adult	3 (0.3%)
Average number of human phenotype ontology (HPO) terms	8.6 ± 4.6
Organ involvement	
Nervous system	1065 (100%)
Musculoskeletal and limb system	767 (71.9%)
Head or neck, including facial dysmorphism	616 (57.8%)
Growth	342 (32.1%)
Eye system	286 (26.8%)
Ear system	230 (21.6%)
Cardiovascular system	200 (18.8%)
Endocrine and metabolism/homeostasis system	185 (17.4%)
Skin	181 (17.0%)
Abnormality of prenatal development or birth	167 (15.7%)
Genitourinary system	146 (13.7%)
Gastrointestinal system	106 (9.9%)
Connective tissue system	72 (6.7%)
Blood and immune system	50 (4.7%)
Respiratory system	35 (3.3%)
Neoplasm	22 (2.1%)
Age at whole exome sequencing	6.5 year (min 0–max 47, SD 8.1)
Total number of patients with previous genetic testing	645 (60.6)
Single gene test	153 (14.4)
Panel test	13 (1.2)
Targeted exome sequencing	20 (1.9)
Chromosome analysis or fluorescence in situ hybridization	355 (33.3)
Microarray	284 (26.6)
Multiplex ligation-dependent probe amplification	103 (9.7)
Mitochondrial full genome sequencing analysis	26 (2.4)

Min, minimum; max, maximum; SD, standard deviation

WES was performed at 6.5 ± 8.1 years (range, 0–47 years). The average number of phenotype items according to the Human Phenotype Ontology (HPO) was 8.6 ± 4.6 per patient. Abnormalities in the nervous system were most frequently observed (96.3% of patients), followed by those in the musculoskeletal system (71.9%), head and neck (57.8%), and growth abnormalities (32.1%) (Table 1).

Of the 1065 patients, 645 (60.6%) underwent separate genetic testing before WES. Furthermore, 20 patients (1.9%) underwent targeted exome sequencing, which included 4813 OMIM genes, and 286 (33. 3%) underwent chromosome microarray. A total of 153 patients (14.4%) underwent single gene testing for monogenic disorders. Other genetic tests included karyotyping and/or fluorescence in situ hybridization (355 patients, 33.3%), multiplex ligation-dependent probe amplification analyses for chromosomal microdeletion or duplication syndromes (103 patients, 9.7%), and mitochondrial full genome sequencing analysis (26 patients, 2.4%). No test revealed a significant positive result.

### Positive results: molecular diagnosis by WES

**Fig. 1:**
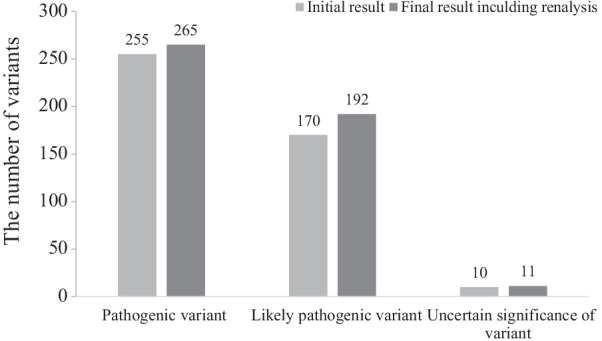
Comparison of the number of pathogenicityof identified variants between the initial and final results, including the reanalysis

**Fig. 2:**
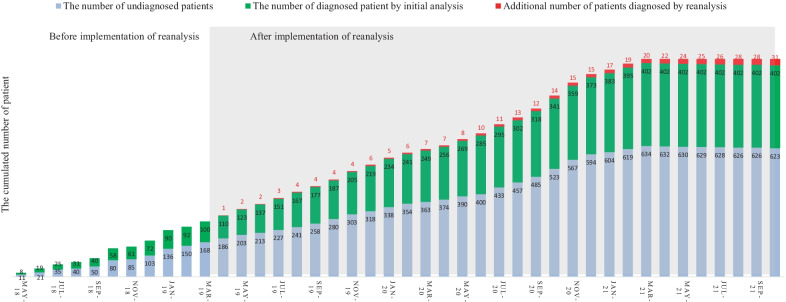
The cumulative monthly number of undiagnosed patients (light blue), patients diagnosed by the initial analysis (green) and by reanalysis (red) from April 2018 to October 2021

**Fig. 3:**
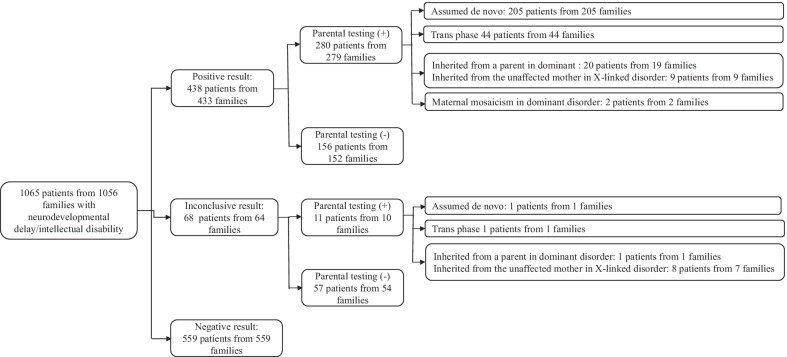
Schematic diagram showing patients divided according to the three reported categories

1. Diagnostic rate including reanalysis
  The clinical and genetic information about the patients with a positive molecular diagnosis is summarized in Additional file 3: Table S3. At initial analysis, 435 variants (255 pathogenic variants, 170 likely pathogenic variants and 10 VUS) were confirmed to be causative in 401 families from 1057 unrelated families (38.0%, 401/1056 families) throughout the study period, April 2018 to August 2021. From April 2019 to October 2021, daily updated reanalysis was performed for patients with no clinically significant variants. This reanalysis identified 49 variants in those 45 patients (Table 2). Among these variants, 34 in31 patients were positively confirmed by reanalysis. Finally, a total of 468 variants (265 pathogenic variants, 192 likely pathogenic variants and 11 VUS) were confirmed to be causative, resulting in the molecular diagnosis of 433 families from 1056 unrelated families (41%, 433/1056 families) (Figs. 1 and 2).
2. Characteristics of variant type and disease
  The most common variant type was missense (45.1%), followed by frameshift (23.2%), stop gain (18.4%), canonical splice site (8.5%), and other (4.8%) variants. Remarkably, 47.5% (220/463) of all confirmed variants were not previously described in any public database.
  Among 433 unrelated patients with a molecular diagnosis, the parents of 279 underwent Sanger sequencing to confirm the segregation pattern of the identified variant. Two hundred three variants were confirmed to be assumed de novo (i.e., biological relationship unconfirmed). Eighty variants from 44 patients were observed in a *trans* pattern with the other variant. Six variants were inherited from symptomatic mothers or fathers. Nine variants of the X chromosome were inherited from asymptomatic mothers. Thirteen variants inherited from an asymptomatic parent were attributed to incomplete penetrance. Two variants showed maternal mosaicism. The number of patients with variants and the inheritance of these variants are summarized in Fig. 3.
  A total of 279 Mendelian disease genes were found in the 433 families, including autosomal dominant (N = 196, 70.3%), autosomal recessive (N = 53, 19.0%), X-linked (N = 24, 8.6%), and autosomal dominant and/or recessive (N = 6, 2.1%) inheritance patterns. Most genes were reported once (202 genes), twice (48 genes) or three times (11 genes). *PTPN11* (Noonan syndrome 1, OMIM 163950) and *ARID1B* (Coffin-Siris syndrome 1, OMIM 135900) were recurrently reported in 11 patients and 10 patients, respectively. Subsequently, *KMT2D* (9 patients), *NF1* (9 patients), *CTNNB1* (7 patients), *ZEB2* (6 patients), *PRRT2* (6 patients), *ANKRD11* (5 patients), *BRAF* (5 patients), *SCN1A* (5 patients), *FOXG1* (5 patients), *ASXL3* (5 patients), *MECP2* (4 patients), *PIK3CA* (4 patients), *EP300* (4 patients), *TUBB3* (4 patients), *WDR45* (4 patients), and *MED13L* (4 patients) were recurrently reported.
3. Dual diagnosis and confirmation of VUSs
  Two patients among the 433 unrelated patients with a positive result received a dual molecular genetic diagnosis. A female patient (ID: 196) showed global developmental delay (GDD), dysmorphic facial features, microphthalmia, micrognathia, leukodystrophy, microcephaly, hypotonia, spasticity, and club foot. She was diagnosed with peroxisome biogenesis disorder 2A (Zellweger) and Mental retardation, X-linked syndromic, Turner type caused by compound heterozygous variants (NM_001300789.1: c.240A > C and c.1622A > G) in the *PEX5* gene and an assumed de novo variant (NM_031407.6: c.12404A > C) in the *HUEW1* gene. A female patient (ID: 98) presenting with hydrocephalus, autistic features, GDD, microcephaly, hypotonia, and dysmorphic facial features was diagnosed with congenital contractures of the limbs and face, hypotonia, and developmental delay, and neurodevelopmental disorder with severe motor impairment and absent language caused by an assumed de novo variant (NM_052867.2:c.3731 T > G) in the *NALCN* gene and a known pathogenic variant (NM_138615.2: c.2344C > T) in the *DHX30* gene, respectively. These genetic variants were associated with either nonoverlapping clinical presentations or contributed to one major phenotype.

**Table 2:** Detailed information on 49 variants in the 45 patients identified by reanalysis

ID	Sex	Gene	Zygosity	HGVS c	Class	Molecular diagnosis	OMIM	Inheritance	Allele origin	Dx	Note
43	F	*TFE3*	Het	NM_006521.6: c.560C > T	P	Intellectual developmental disorder, X-linked, syndromic, with pigmentary mosaicism and coarse facies	301066	XL	Unknown	Yes	New disease
59	M	*SPEN*	Het	NM_015001.3: c.5806C > T	P	Radio-Tartaglia syndrome	619312	AD	Assumed de novo	Yes	New disease
62	M	*ADH5*	Het	NM_000671.4:c.966del	P	AMED syndrome, digenic	619151	AR	Unknown	Yes	New disease
86	F	*GABRG2*	Het	NM_198903.2:c.316G > A	P	Epileptic encephalopathy, early infantile, 74	618396	AD	Assumed de novo	Yes	New disease
90	F	*MSL3*	Het	**NM_078629.3:c.1226_1229del**	P	Basilicata-Akhtar syndrome	301032	XL	Assumed de novo	Yes	New disease
92	F	*TFE3*	Het	NM_006521.5: c.559A > G	LP	Intellectual developmental disorder, X-linked, syndromic, with pigmentary mosaicism and coarse facies	301066	XL	Assumed de novo	Yes	New disease
99	M	*MAPK8IP3*	Het	NM_001318852.1: c.1735C > T	P	Neurodevelopmental disorder with or without variable brain abnormalities	618443	AD	Assumed de novo	Yes	New disease
138	F	*LTBP1*	Het	**NM_206943.2:c.1342C > T**	LP	Cutis laxa, autosomal recessive, type IIE	619451	AR	Unknown	Yes	New disease
		*LTBP1*	Het	**NM_206943.2: c.4793_4794del**	LP				Unknown		
206	M	*PTPN23*	Het	**NM_015466.3: c.345del**	LP	Neurodevelopmental disorder and structural brain anomalies with or without seizures and spasticity	618890	AR	Unknown	Yes	New disease
		*PTPN23*	Het	NM_015466.3:c.4052A > G	VUS		618890	AR	Unknown		
275	F	*TFE3*	Het	**NM_006521.5: c.569A > G**	LP	Intellectual developmental disorder, X-linked, syndromic, with pigmentary mosaicism and coarse facies	301066	XL	Unknown	Yes	New disease
345	M	*DLG4*	Het	**NM_001365.4:c.1608-2A > G**	LP	Intellectual developmental disorder 62	618793	AD	Unknown	Yes	New disease
399	F	*MAP1B*	Het	**NM_005909.4:c.6715del**	LP	Periventricular nodular heterotopia 9	618918	AD	Unknown	Yes	New disease
434	M	*EMC10*	Het	NM_206538.3:c.343C > T	LP	Neurodevelopmental disorder with dysmorphic facies and variable seizures	619264	AR	Unknown	Yes	New disease
		*EMC10*	Het	NM_206538.3:c.70C > T	LP		619264	AR	Unknown		
517	M	*ZNF292*	Het	**NM_015021.3:c.6015dup**	LP	Intellectual developmental disorder, autosomal dominant 64	619188	AD	Unknown	Yes	New disease
532	M	*ZMYM2*	Het	**NM_001190964.4:c.3472C > T**	LP	Neurodevelopmental-craniofacial syndrome with variable renal and cardiac abnormalities	619522	AD	Unknown	Yes	New disease
536	F	*PHF21A*	Het	**NM_001101802.1:c.1171A > T**	P	Intellectual developmental disorder with behavioral abnormalities and craniofacial dysmorphism with or without seizures	618725	AD	Assumed de novo	Yes	New disease
548	F	*ALKBH8*	Hom	NM_001301010.1:c.1430_1438del	LP	Intellectual developmental disorder, autosomal recessive 71	618504	AR	Trans phase	Yes	New disease
570	M	*TET3*	Het	**NM_001287491.1:c.2161_2183dup**	P	Beck-Fahrner syndrome	618798	AD/AR	Assumed de novo	Yes	New disease
604	M	*SETD1B*	Het	**NM_015048.1:c.5508dup**	LP	INTELLECTUAL DEVELOPMENTAL DISORDER WITH SEIZURES AND LANGUAGE DELAY; IDDSELD	619000	AD	Unknown	Yes	New disease
656	F	*SATB1*	Het	NM_001195470.2:c.1574A > G	LP	Kohlschutter-Tonz syndrome-like	619229	AD	Assumed de novo	Yes	New disease
709	F	*RALA*	Het	**NM_005402.3:c.404_429dup**	LP	Hiatt-Neu-Cooper neurodevelopmental syndrome	619311	AD	Unknown	Yes	New disease
752	M	*KCNMA1*	Het	NM_001271519.1:c.2423C > T	LP	Liang-Wang syndrome	618729	AD	Unknown	Yes	New disease
776	F	*NCDN*	Het	**NM_001014839.1:c.990dup**	LP	Neurodevelopmental disorder with infantile epileptic spasms	619373	AD/AR	Unknown	Yes	New disease
792	M	*GRIA2*	Het	**NM_001083619.1:c.1957_1958insT**	LP	Neurodevelopmental disorder with language impairment and behavioral abnormalities	618917	AD	Unknown	Yes	New disease
1051	F	*SETD1A*	Het	NM_014712.2:c.4582-2_4582-1del	LP	Neurodevelopmental disorder with speech impairment and dysmorphic facies	619056	AD	Unknown	Yes	New disease
1053	F	*ZNF292*	Het	**NM_015021.3:c.3862dup**	LP	Intellectual developmental disorder, autosomal dominant 63	619188	AD	Unknown	Yes	New disease
116	M	*KCNT2*	Het	NM_198503.3:c.2501_2507del	LP	Developmental and epileptic encephalopathy 57	617771	AD	Unknown	Yes	Variant update
196	F	*HUEW1*	Het	NM_031407.6:c.12404A > C	LP	Mental retardation, X-linked syndromic, Turner type	309590	XL	Assumed de novo	Yes	Variant update
286	M	*KMT2D*	Het	NM_003482.3:c.10744C > T	P	Kabuki syndrome 1	147920	AD	Unknown	Yes	Variant update
379	M	*HK1*	Het	NM_001322365.1:c.901G > A	LP	Neurodevelopmental disorder with visual defects and brain anomalies	618547	AD	Unknown	Yes	Variant update
354	F	*ACTB*	Het	NM_001101.3:c.124G > A	LP	Baraitser-Winter syndrome 1	243310	AD	Assumed de novo	Yes	Clinical update
411	F	*TRPM3*	Het	NM_001007471.2:c.2968G > A	P	TRPM3-related intellectual disabilities with epilepsy	PMID 31278393	AD	Unknown	Not yet	Candidate gene
1052	M	*TMPRSS9*	Het	NM_182973.2:c.1094C > A	LP	TMPRSS9-related autism spectrum disorder	PMID 31943016	AR	Unknown	Not yet	Candidate gene
120	F	*CHRM1*	Het	NM_000738.2:c.1274 T > C	LP	CHRM1-related neurodevelopmental disorder	PMID 34212451	AD	Unknown	Not yet	Candidate gene
105	F	*ATAD3A*	Het	NM_018188.3:c.1879C > T	VUS	Pontocerebellar hypoplasia, hypotonia, and respiratory insufficiency syndrome, neonatal lethal	618810	AR	Unknown	Not yet	New disease
		*ATAD3A*	Het	NM_018188.3:c.1586G > A	VUS		618810	AR	Unknown		New disease
39	M	*CUL3*	Het	NM_003590.5:c.383G > A	VUS	Neurodevelopmental disorder with or without autism or seizures	619239	AD	Unknown	Not yet	New disease
703	F	*POLR2A*	Het	**NM_000937.4:c.1832A > T**	VUS	Neurodevelopmental disorder with hypotonia and variable intellectual and behavioral abnormalities	618603	AD	Unknown	Not yet	New disease
860	F	*USP9X*	Het	**NM_001039590.3:c.4796 T > C**	VUS	Mental retardation, X-linked 99, syndromic, female-restricted	300968	XD	Unknown	Not yet	Variant update
885	F	*KDM1A*	Het	NM_001009999.2:c.1901C > T	VUS	Cleft palate, psychomotor retardation, and distinctive facial features	616728	AD	Unknown	Not yet	Variant update
25	M	*CHD7*	Het	**NM_017780.3:c.2499-11 T > A**	VUS	CHARGE syndrome	214800	AD	Unknown	Not yet	Variant update
67	M	*FGFR1*	Het	**NM_001174067.1:c.1099G > A**	VUS	Osteoglophonic dysplasia	166250	AD	Unknown	Not yet	Variant update
514	F	*EBF3*	Het	**NM_001005463.3:c.373G > A**	VUS	Hypotonia, ataxia, and delayed development syndrome	617330	AD	Unknown	Not yet	Variant update
902	F	*SPTBN1*	Het	**NM_003128.2:c.5708G > A**	VUS	Developmental delay, impaired speech, and behavioral abnormalities	619475	AD	Unknown	Not yet	Variant update
979	F	*SPTBN1*	Het	**NM_003128.3:c.2471 T > C**	VUS	Developmental delay, impaired speech, and behavioral abnormalities	619475	AD	Unknown	Not yet	Variant update
990	M	*NSF*	Het	**NM_006178.4:c.1359G > A**	VUS	Developmental and epileptic encephalopathy 96	619340	AD	Unknown	Not yet	Variant update

P, pathogenic; LP, Likley pathogenic; VUS, uncertain significance of variant; AD, autosomal dominant; AR, autosomal recessive; XL, X-linked; heterozygous, Het; homozygous, Hom; Dx, diagnosis; Bold: novel variant

Among the 72 VUSs identified in 68 patients that were determined to involve clinically relevant genes based on OMIM data or recent PubMed publications, positive results were obtained for 11. Six of these 11 VUSs were confirmed to be in trans phase with other LP or P variants in genes associated with autosomal recessive disorder and were highly matched with disease-relevant symptoms. Three VUSs (ID: 206, 210, and 697) with other LP or P variants also showed highly specific phenotypes associated with each disease; however, phase status was undetermined due to a lack of familial segregation analysis. Importantly, the VUS of 2 patients was confirmed by clinicians using other genetic testing methods and by assessing treatment responses. A male patient (ID: 143) had maternal UPD 9 encompassing a region containing the VUS (Seo et al. 2020). A male patient (ID: 71) with a homozygous variant in the *PREPL* gene responded to medical treatment after molecular diagnosis (Kim et al. 2020).

### Inconclusive results

Sixty-four variants, including 1 P, 2 LP and 61 heterozygous VUSs, were assessed as inconclusive, i.e., insufficient to be responsible for the patients’ phenotypes. Fifty-five variants had not been previously reported. These variants were identified in 61 patients from 57 unrelated families (Additional file 4: Table S4) and associated with 36 autosomal dominant, 6 autosomal recessive and 12 X-linked disorders. Furthermore, we identified 1 assumed de novo LP variant and 7 heterozygous variants in 7 genes that have not been registered as OMIM morbid genes but have been described in a few studies (Additional file 4: Table S4).

### Summary of reanalyzed results and description of patients diagnosed with a new disease

**Fig. 4:**
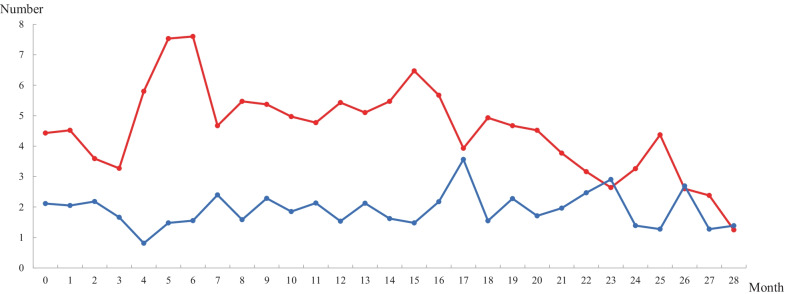
The number of patients with newly reclassified variants (red line) and the number of newly reclassified variants (blue line) based on daily reanalysis data from April 2019 to October 2021

**Fig. 5:**
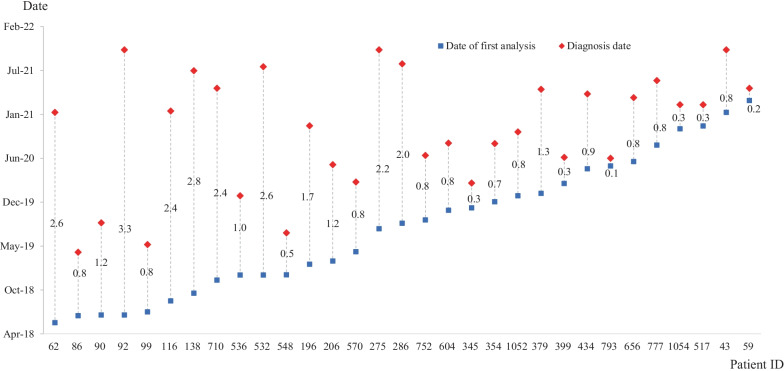
The time interval between the first analysis (blue dot) and the molecular diagnosis (red dot) of patients diagnosed by reanalysis. Number: Time interval converted to years

From April 2019 to October 2021, daily updated reanalysis reported an average of 2.1 ± 0.7 newly reclassified variants in an average of 4.1 ± 1.7 patients per day among all undiagnosed patients (Fig. 4). The time interval between the first analysis and the molecular diagnosis by reanalysis was 1.2 ± 0.9 years (from a minimum of 1 month to a maximum of 3.3 years, Fig. 5). This time interval was shorter among the patients enrolled during the latter part of the study period. Table 2 shows 49 variants identified in the 45 patients by reanalysis (Table 2). Diagnosis by reanalysis was possible in 26 patients due to the discovery of a new gene-disease relationship. They were diagnosed with 23 different diseases that were discovered after 2019. In addition, the updated variant reclassification and updated phenotype description by physicians helped diagnose 4 patients and 1 patient, respectively. Molecular diagnoses by reanalysis were achieved for 31 patients. In addition, the results were inconclusive in the remaining 14 patients (1.3%, 14/1056) with 2 P/LP variants in 2 candidate genes and 12 VUSs due to lack of additional in vivo or in vitro evidence.

In 6 patients, the diagnosis was obtained by automated daily updated analysis approximately 100 days after the initial report, e.g., in a male patient (ID: 59) who presented with hypotonia, facial dysmorphism including thin ear helix, prominent ear, prominent upper lip vermilion, short philtrum, bulbous nose, flat nasal root, hypertelorism and thick eyebrow, hearing loss, optic atrophy, nephrocalcinosis, autistic features, and global developmental delay. The patient’s initial March 2021 report was negative; however, a *SPEN* variant was first reported in March 2021 in 13 individuals with similar phenotypes (Radio et al. 2021). On May 3, 2021, this variant was registered as a new entry associated with Radio-Tartaglia syndrome (OMIM 619312). The patient was found to have the P variant in *SPEN* (NM_015001.3: c.5806C > T). Therefore, 2 months after the initial report, he was diagnosed with Radio-Tartaglia syndrome (OMIM 619312) by reanalysis. A male patient (ID: 345) presented with intellectual disability, autistic features, periventricular leukomalacia, and facial dysmorphism, including prominent ears, flat nasal root, prominent upper vermilion, and hypertelorism. The initial report was negative in Nov 2019. On Feb 26, 2020, intellectual developmental disorder 62 (OMIM 618793) caused by the *DLG4* variant was registered as a new entry. This patient was found to have the LP variant in *DLG4* (NM_001365.4: c.1608-2A > G). Therefore, 3 months after the initial report, he was diagnosed with intellectual developmental disorder 62 (OMIM 618793) by reanalysis. A female patient (ID: 399) presented with GDD, heterotopia, failure to thrive, seizures, and facial dysmorphism. She had the LP variant in *MAP1B* (NM_005909.4: c.6715del), but the initial report was inconclusive in Feb 2020 since this gene-phenotype association had been reported but not yet registered in OMIM (Julca et al. 2019). On June 22, 2020, periventricular nodular heterotopia 9 (OMIM 618918) caused by the *MAP1B* variant was registered as a new entry. Therefore, 3 months after the initial report, this patient was diagnosed with periventricular nodular heterotopia 9 (OMIM 618918) by reanalysis. A male patient (ID: 793) presented with GDD, strabismus, and facial dysmorphism. He had the LP variant in *GRIA2* (NM_000826.6: c.1958_1960delCCAinsTCTACAGCAC), but the initial report was inconclusive in May 2020 since this gene-phenotype association was reported in July 2019 but had not been registered in OMIM (Salpietro et al. 2019). On June 18, 2020, neurodevelopmental disorder with language impairment and behavioral abnormalities (OMIM 618917) caused by the *GRIA2* variant was registered as a new entry. Therefore, 1 month after the initial report, the patient was diagnosed with neurodevelopmental disorder with language impairment and behavioral abnormalities (OMIM 618917) by reanalysis. Finally, one male (ID: 517) and one female (ID: 1054) patient presenting with GDD received negative initial reports in December 2020. The *ZNF292* variant was first reported in 28 families with intellectual disabilities in March 2020 (Mirzaa et al. 2020). On February 2021, this variant was registered as a new entry associated with intellectual developmental disorder, autosomal dominant 64 (OMIM 619188). The two patients had LP variants in *ZNF292* (NM_015021.3: c.6015dup and NM_015021.3: c.3862dup). Therefore, 2 months after the initial report, they were diagnosed with intellectual developmental disorder, autosomal dominant 64 (OMIM 619188), by reanalysis.

### Clinical actionability after diagnosis

The genetic diagnosis changed the clinical management in 4.0% of the diagnosed patients (16/402 pts) (Table 3). After diagnosis, eight patients with seizure disorder (ID: 35, 228, 269, 285, 314, 442, 518, and 519) changed antiepileptic drug. Ketogenic diet was started in a patient 159 with GLUT1 deficiency syndrome 1. Patient 71 and 363 with myasthenic syndrome, congenital, 22 and segawa syndrome, recessive received pyridostigmine and levodopa beginning at age 2.6 years and 10 months, respectively (Kim et al. 2020). Patient 278 and 402 with urea cycle disorder were managed with low protein diet. Patient 229 and 383 with neurofibromatosis type 1 having inoperable plexiform neurofibroma was treated with selumetinib as in a clinical trial (https://cris.nih.go.kr/cris/search/detailSearch.do/19080). In case of patient 257, dystonia was refractory to medical treatment, and deep brain stimulation surgery was performed.

**Table 3:** The 16 patients in whom clinical management was changed after the genetic diagnosis

Patient ID	Gene	Disease	Medical treatment
35	*SCN8A*	Epileptic encephalopathy, early infantile, 13	Na channel blocker incluidng oxcarbazepine and phenytoin
71	*PREPL*	Myasthenic Syndrome, Congenital, 22	Pyridostigmine
159	*SLC2A1*	GLUT1 Deficiency Syndrome 1	Ketogenic diet
228	*SCN8A*	Epileptic encephalopathy, early infantile, 13	Na channel blocker incluidng oxcarbazepine and phenytoin
269	*KCNQ2*	Epileptic encephalopathy, early infantile, 7	Na channel blocker incluidng oxcarbazepine and phenytoin
278	*CPS1*	Carbamoylphosphate synthetase I deficiency	Low protein diet
285	*SCN1A*	Epileptic encephalopathy, early infantile, 6	Valprotic acid, topiramate
314	*SCN2A*	Epileptic encephalopathy, early infantile, 11	Valprotic acid, topiramate
363	*TH*	Segawa syndrome, recessive	Levodopa
402	*ASS1*	Citrullinemia	Low protein diet
442	*KCNQ2*	Epileptic encephalopathy, early infantile, 7	Na channel blocker incluidng oxcarbazepine and phenytoin
518	*PRRT2*	Seizures, benign familial infantile, 2	Oxcarbazepin
519	*SCN1A*	Febrile seizures, familial, 3A	Valprotic acid, topiramate
229	*NF1*	Neurofibromatosis Type 1	Selumetinib
257	*TOR1A*	Dystonia-1, torsion	Deep brain stimulation
288	*ARSA*	Metachromatic leukodystrophy	Bone marrow transplantation candidate
383	*NF1*	Neurofibromatosis Type 1	Selumetinib

### Secondary findings

Forty-two variants, including 16 P and 19 LP variants, were identified in the 17 genes recommended to be reported as secondary findings by the ACMG guidelines (v2.0) in 35 of the 1056 patients (3.3%). Twenty-nine variants (16 P and 13 LP variants) had been previously reported to be P or LP. Pathogenic variants in the *BRCA2* gene were the most commonly identified, but recurrent variants were not detected. Genetic counseling was provided for the patients and their family members, and appropriate surveillance was conducted depending on the identified genes.

## Discussion

WES is an effective diagnostic approach with a higher diagnostic rate than gene panels or CMA (Clark et al. 2018; Srivastava et al. 2019). Studies employing WES for investigating neurodevelopmental disorders have revealed that the diagnostic yield varies from 30 to 50% (Lecoquierre et al. 2019; Clark et al. 2018; Ligt et al. 2012; Kim et al. 2019). The diagnostic yield of ~ 40% found here indicates that WES is a valuable tool for diagnosing patients with NDD/ID and is comparable to that observed in other studies.

Most of our patients had syndromic features with an average of 8 organ system abnormalities, indicating widely variable phenotypic heterogeneity. In line with these clinical observations, molecular diagnosis revealed a wide range of genetic diseases associated with P/LP variants in 279 different Mendelian disorder genes. Most genes were reported once (202 genes) or twice (48 genes), and only a small portion of genes (30 genes) were recurrently reported in our study.

In previous retrospective studies, reanalysis has increased the diagnostic rate of 10% over a period of 18–36 months, with total diagnostic rate as ~ 40% (Fung et al. 2020). In our study, the diagnostic rate was initially 38.0%, and reanalysis increased the diagnostic rate by 3%, which was lower than the increase observed in previous studies (Liu et al. 2019; Costain et al. 2018; Fung et al. 2020; Wenger et al. 2017). The main reason for this lower enhancement is that our automated system was updated daily and applied the new information to the analysis immediately after each new gene-phenotype causality was reported. As in the prospective study, this daily update allowed diagnoses based on the initial analyses of patients enrolled in the latter part of the study. Indeed, the interval between the date of ordering for WES and the date of the gene-disease causality was originally reported was less than 1 year in the 17 identified diseases. As shown in Fig. 2, the relatively consistent diagnostic rate enhancement by reanalysis reflects the effect of daily updates of the analysis system. In addition, this time interval was shorter among the patients enrolled during the latter part of the study period, as shown in Fig. 5, demonstrating the excellent performance of this automated, daily reanalysis approach.

It has recently been recommended that reanalysis of an individual’s genome or exome sequencing data should be performed every 1–2 years until diagnosis, or more frequently if their phenotype evolves, considering the cost and resources used (Costain et al. 2018; Ewans et al. 2018). However, it is not easy or convenient to regularly check up on and reanalyze every undiagnosed patient, and updated diagnoses can be easily missed. Our automated daily updated analysis system can effectively reanalyze data from undiagnosed patients, minimizing missed diagnoses.

A variety of reasons for the improved diagnostic yield obtained by reanalysis have been reported, including new gene–disease associations and literature updates, updated and clarified patient phenotypes, additional sequencing for trios/other affected individuals and family members, improvements in sequencing data by resequencing strategies, upgraded bioinformatics tools, and research collaborations (Liu et al. 2019; Fung et al. 2020; Won et al. 2020). The main reason for obtaining additional diagnoses by reanalysis has been attributed to the discovery of new gene-disease associations (Liu et al. 2019; Fung et al. 2020). Approximately 250 novel gene–disease and 9200 novel variant–disease associations are reported yearly (Wenger et al. 2017). New entries are uploaded daily to OMIM or ClinVar, which is one of the most important databases of diseases and variants. In the current study, most of the reanalyzed patients were diagnosed due to newly discovered gene-disease associations. Six patients were diagnosed with a new disease within approximately 100 days of the first analysis. In addition, among two patients with dual molecular genetic diagnoses, one patient (ID: 196) was initially diagnosed with peroxisome biogenesis disorder 2A. However, the patient’s data were reanalyzed due to compound heterozygous variants including one LP variant and one VUS of the *PEX5* gene. One VUS of the *HUEW1* gene with high similarity scores were selected, leading to a dual genetic diagnosis as it was confirmed to be assumed de novo. Therefore, the reanalysis system can be effective, identifying a new diagnosis in a patient who was previously clinically diagnosed but with molecularly inconclusive results.

Reanalysis is difficult in conventional clinical laboratories because it is time-consuming and labor-intensive. In addition, frequent changes in analytical pipelines may hinder routine work. In our reanalysis system, an average of 2.1 variants per patient were newly reclassified in an average of 4 undiagnosed patients daily up to 800 days after the initial analysis. Since variants that were newly uploaded and reviewed the previous day were excluded from the subsequent analysis, the number of newly reclassified variants tended to decrease after 800 days. Therefore, an automated pipeline-based daily updated system significantly reduced the workload required for the periodic reanalysis of undiagnosed patients.

Several studies comparing the diagnostic yield of proband-only WES with trio WES in patients with a complex phenotype have shown that trio WES provides an incremental gain of 10–15%, mainly because de novo variants can be easily identified (Srivastava et al. 2019; Tan et al. 2019). Moreover, the better efficiency of trio WES versus proband-only WES was attributed to the nearly twofold reduction in the number of variants selected for curation due to the use of parental information (Tan et al. 2019). Although candidate variants identified by proband-only WES require additional targeted Sanger sequencing to confirm segregation, information that is intrinsically obtained from trio WES, the overall diagnostic yield of 41% in this study using proband-only WES was in line with previous studies of trio WES for patients with NDD/ID (Clark et al. 2018; Srivastava et al. 2019). A trio-based approach has been more effective in discovering novel gene-disease associations and in increasing confidence in the role of variants of unknown significance (Bertoli-Avella et al. 2021; Farwell et al. 2015). However, in a clinical setting, trio testing would not be feasible due to the increased costs of parental WES and the possibility that parental DNA is not available. In addition, the curating times and costs of WES analysis were reduced due to automatic variant interpretation by EVIDENCE (Seo et al. 2020; Kim et al. 2021), thus suggesting that proband-only WES by EVIDENCE is a comparable diagnostic approach for these patients in a routine clinical setting.

This study had several limitations. First, family member testing could not be performed in 279 of the 433 patients diagnosed with genetic diseases due to unavailable samples. Second, maternity and paternity confirmation was not performed due to Confucian ideals of South Korea. Most de novo occurrences were assumed. Third, the classification of variants may change as information is updated in the daily-updated databases. Therefore, as criteria for each variant can be added or removed based on updated information, the variant classification change. Fourth, not all patients with inconclusive or negative results were reanalysed, and only those with variants newly reclassified by automatic reanalysis were rereviewed. However, there may be differences in variant classification depending on how the cutoff criteria for selecting a variant are set based on the ACMG guidelines and additional databases. In addition, symptom similarity may be underestimated if information regarding disease or patient symptoms is limited. Finally, since a manual review was not performed for the variants not selected by the automatic reanalysis system, it is possible that the diagnosis was missed in some patients.

## Conclusion

In conclusion, this study was the first to consider the diagnostic and clinical utility of WES in a large, multimedical center cohort group of patients with NDD/ID in South Korea. Our results suggest that proband-only WES could be useful for the genetic diagnosis of patients with NDD/ID with diverse genetic causes. Daily updated databases and reanalysis systems can provide a higher diagnostic rate using initial analysis and contribute to the rapid diagnosis of undiagnosed patients by reflecting the latest medical knowledge.

## Supplementary Information

**Additional file 1: Table S1. **Sequencing quality information for 1065 patient.

**Additional file 2: Table S2. **Database used for variant annotation and interpretation.

**Additional file 3: Table S3. **Detailed information on patients with a positive result.

**Additional file 4: Table S4. **Detailed information on patients with inconclusive result.

## Data Availability

All variant and phenotype data supporting findings of this study are available within the manuscript and supplement tables. The pathogenic variants identified in this study have been submitted to ClinVar with Accession Numbers (SCV002011902-SCV002012361, SCV002011867, SCV002011868, SCV002011869, and SCV002011870). The raw data of whole-exome sequencing of the patient in this study are not publicly available to protect participant confidentiality, but they are available from the corresponding author on reasonable request.

## Abbreviations

WES: Whole-exome sequencing
NDD: Neurodevelopmental delay
ID: Intellectual disability
GDD: Global developmental delay
CMA: Chromosomal microarray
ACMG: American College of Medical Genetics
VUS: Uncertain significance of variant
P: Pathogenic
LP: Likely pathogenic
HPO: Human Phenotype Ontology
